# Emergency care via video consultation: interviews on patient experiences from rural community hospitals in northern Sweden

**DOI:** 10.1186/s12245-024-00703-4

**Published:** 2024-09-03

**Authors:** Lina Ärlebrant, Hanna Dubois, Johan Creutzfeldt, Anette Edin-Liljegren

**Affiliations:** 1 Department of Epidemiology and Global Health, Umeå University, Centre for Rural Health, Region Västerbotten, Storuman, Sweden; 2 Department of Clinical Science and Education, Södersjukhuset, Karolinska Institutet, Stockholm, Sweden; 3 Department of Clinical Science, Intervention and Technology, Karolinska Institutet, Stockholm, Sweden; 4 Department of Epidemiology and Global Health, Umeå University, Centre for Rural Health, Region Västerbotten, Storuman, Sweden

**Keywords:** Emergency care, Video consultation, Patient experience, Qualitative, Rural, Telehealth, Emergency nursing, Emergency nurse, General practitioner, Community hospital

## Abstract

**Background:**

Delivering emergency care in rural areas can be challenging, but video consultation (VC) offers opportunities to make healthcare more accessible. The communication and relationship between professionals and patients have a significant impact on the patient’s experience of safety and inclusion. Understanding the patient perspective is crucial to developing good quality healthcare, but little is known about patient experiences of emergency care via VC in a rural context. The aim of this study was to explore patient experiences of emergency care via VC in northern rural Sweden.

**Methods:**

Using a qualitative approach, semi- structured interviews (*n* = 12) were conducted with individuals aged 18—89 who had received emergency care with a registered nurse (RN) on site and VC with a general practitioner (GP). The interviews were conducted between October 2021 and March 2023 at community hospitals (*n* = 7) in Västerbotten County, Sweden. Interviews were analysed with content analysis.

**Results:**

The analysis resulted in main categories (*n* = 2), categories (*n* = 5) and subcategories (*n* = 20). In the main category, “We were a team of three”, patients described a sense of inclusion and ability to contribute. The patients perceived the interaction between the GP and RN to function well despite being geographically dispersed. Patients highly valued the opportunity to speak directly to the GP. In the main category, “VC was a two-sided coin”, some experienced the emergency care through VC to be effective and smooth, while some felt that they received a lower quality of care and preferred face-to-face consultation with the GP. The quality of the VC was highly dependent on the RN’s ability to function as the hub in the emergency room.

**Conclusion:**

Patients in rural areas perceived being included in 'the team' during VC, however they experienced disadvantages with the system on individual basis. The nursing profession plays an important role, and a proper educational background is crucial to support RNs in their role as the hub of the visit. The GP’s presence via VC was seen as important, but to fully enable them to fulfil their commitments as medical professionals, VC needs to be further improved with education and support from technical devices.

**Supplementary Information:**

The online version contains supplementary material available at 10.1186/s12245-024-00703-4.

## Introduction

Health is a fundamental human right central for a sustainable development with universal health coverage where “all people- no matter who they are or where they live- can receive quality health services” [1]. However, there is still persisting inequalities in access to essential health services for population groups living in rural settings [1]. To improve health coverage, WHO has presented the global strategy on digital health and acknowledge that appropriate use of digital technologies should be contextualized and align countries to ensure that quality and outcome are assessed on new health technologies and shared across domains[2]. The use of telehealth service has been widely applied internationally improving access to health services for patients regardless of their setting. In contrast, increasing evidence show that telehealth is not suitable for all patients due to its highly inaccessible format to persons with disabilities [3] and needs to be evaluated for whom, when, how, and where.

Video consultation (VC) is an application of telehealth that provides real-time visual and audio connection [4] and creates opportunities to deliver medical support to areas where it is lacking. Thus, a great deal of responsibility for care and treatment, particularly in rural areas, has been moved from the hospital to primary care units [5].

Depending on the definition, roughly three quarters of all US landmass can be classified as rural [6], and Australia, Europe, Canada and Africa also have large areas with populations < 1 inh.^/km2^[7]. When distances are great between healthcare facilities, patients sometimes have fewer options and limited access to quality care. VC can reduce the need to travel and provides patients with better access to the healthcare they need. The role that VC should play in overcoming inequalities in healthcare access, and the degree to which it may actually contribute to these inequalities, is an unanswered question that urgently needs to be addressed [8, 9].

Systematic reviews investigating patient satisfaction with telehealth, such as VC indicate that VC has an overall positive impact on patient satisfaction [10, 11]. However, several studies show that patients and healthcare professionals would prefer a face-to-face consultation to a VC if given the choice [11–13]. When comparing content and quality between VC and telephone used in consultations in primary care, both have similar outcomes, though VC may offer some advantages, for example, in respect of building rapport [14]. There is growing evidence suggesting that to improve healthcare, patient involvement is needed [2, 15, 16]. Also in studying the effectiveness of telemedicine, patient perspectives is pointed out as an area in need of more knowledge [17].

Increasing access to technology such as video conference equipment has changed the nature of encounters between patients and healthcare professionals. However, there is still limited knowledge about patients’ views on this new form of care [18]. We therefore need more knowledge about VC in healthcare, as this communication solution is increasingly used, especially in a rural context. Straight-forward and continuous communication between professionals and patients has been shown to be an essential aspect of patient involvement in emergency care visits [19]. Concerning patients’ experiences with specialist care via VC in rural primary healthcare, research has found that good communication is essential for the patients’ sense of safety during the VC encounter [20]. The patient–healthcare professional relationship, especially with the local registered nurse (RN), has been highlighted as another important aspect in a rural eHealth context [21]. However, rural practitioners often have insufficient training or a background that is less relevant to rural practice and context [22]. In the literature, the most common VC scenario in emergency care is when a rural, small hospital connects with an urban emergency department in a consultation between two physicians [20, 23]. The use of VC in emergency care has also been investigated for certain acute medical conditions, such as psychiatric emergencies [24] and acute stroke [25, 26] but few studies have explored emergency care via VC in a rural primary care setting [27, 28]. In northern rural Sweden the residents have access to primary care at 7 small community hospitals and emergency care with a general practitioner (GP) via VC, which has not been evaluated.

### Aim

The aim of this study was to explore patient experiences of emergency care via VC at community hospitals in northern rural Sweden.

## Methods

### Study design

This study has a qualitative design with an inductive approach [29]. Semi-structured, individual interviews were carried out with patients and analysed using content analysis as described by Graneheim and Lundman [30]. Reporting of this study follows the Consolidated criteria for reporting qualitative research (COREQ) [31].

### Setting

The study was performed in northern Sweden in the county of Västerbotten. Northern rural Sweden has a climate with long, cold winters characterized by heavy snowfall and freezing temperatures. For the population living here, journeys of 350 km to see a specialist clinician are not uncommon [32]. Rural municipalities in western Västerbotten County have a population density of 0.78/km^2^ and access to primary care and acute care service in 7 smaller community hospitals staffed with GPs, RNs, district nurses, physiotherapists, occupational therapists, midwives and assistant nurses [33]. The community hospitals have an inpatient ward with 2–8 beds and plain X-ray. In the hospital ward GPs are responsible for treating a variety of acute and post-acute conditions as well as end-of- life care [33]. There is no international definition of a community hospital but one definition applicable for the Swedish context is a hospital where “the admission, care and discharge of patients are under the direct control of a GP” [34]. During on-call hours, the acute care service includes ambulance transportation to the emergency rooms in the community hospitals which is staffed with a RN, whereas a GP can be consulted via video equipment [35]. A nursing assistant can provide reinforcement in case of a severe emergency [27]. On-call hours, one GP is responsible for several community hospitals and can be consulted over telephone or via video [36]. The emergency care investigated in this study was provided during on-call hours in the emergency room at community hospitals. None of the patients in this study had called an ambulance for transportation to the community hospital, but arranged with transportation themselves.

### Participants

Participants were selected using convenience sampling. The RNs responsible for the emergency room service in the community hospitals recruited the participants. The inclusion criteria were adult patient (> 18 years) receiving unscheduled emergency care at one of the 7 community hospitals within 3 months of interview and able to remember it, and able to speak Swedish. Patients who expressed an interest in participation were asked to write down their contact details and received a letter with written information about the study. The first author contacted potential participants by telephone to provide verbal information about the study and schedule the interview. Participants could choose to conduct the interview face-to-face, in a video meeting or by telephone. All participants chose to be interviewed by telephone. Participants were encouraged to seek a quiet place without much interruption during the interview. A total of 19 patients agreed to be contacted about the study, and 12 were included and interviewed (Table 1). Seven potential patients were excluded: three did not answer the phone despite repeated attempts, and too much time had passed between VC and contact for four potential patients (more than 3 months). The time between emergency care via VC and the interview varied for most participants between 6–14 days, but for one participant, the time was 3 months. The sample size was motivated using the concept “information power” instead of data saturation, since the method of analysis were qualitative content analysis, the study aim was narrow and the informants had characteristics highly specific for the aim [37].

**Table 1:** Participant characteristics

Variable	Interviewed *n* = 12 (%)
**Age**	18–89 years
**Female sex**	9 (75%)
**Male sex**	3 (25%)
**Born and raised in Sweden**	10 (83.3%)
**Born and raised in other country**	2 (16.7%)
**Cause of care contact**	
Injured in machine	1 (8.3%)
Injured by animal	2 (16.7%)
Cut wound	3 (25%)
Heart fibrillation	1 (8.3%)
Pain in mouth/throat	2 (16.7%)
Pain in eye	2 (16.7%)
Unconsciousness	1 (8.3%)
**Digital competence**	
High	1 (8.3%)
Intermediate	10 (83%)
Low	1 (8.3%)

### Data collection

The data collection was conducted from October 2021 to March 2023. An interview guide was developed by the research team (LÄ, AEL, HD, JC), which has extensive research experience in rural areas, including remote healthcare, interviewing, medicine and nursing. The interview guide (Appendix 1.) focused on reception, involvement, safety and interaction with remote healthcare staff. After the first interview, the interview guide were evaluated in the research team which found it to work satisfyingly. The interviews were also continuously evaluated concerning information gain, and the authors saw no need to revise the interview guide. The semi-structured, individual interviews were carried out by the first author, who does not have a care relationship with any of the participants in this study but was distantly acquainted with one of them. The other authors only had access to anonymized data material and did not have any relationship with any of the participants. The interviews lasted between 17—62 min. The interviews were recorded and transcribed verbatim.

### Data analysis

The data analysis was carried out by the first author in consultation with the co-authors (AEL, HD), using MAXQDA software (Version: MAXQDA 2022 (Release 22.8.0)). The analysis process followed the steps and concepts of content analysis described by Graneheim and Lundman [38]. Transcripts were read through several times to get a sense of the entire data. The elements of the text that related to the aim of the study were marked as meaning units. These units were condensed and given codes that describe the content. Codes that share content were grouped in sub-categories, and the sub-categories were further sorted and abstracted into categories that expressed the manifest content of the text (Table 2). Movement back and forth between the whole transcripts and the coding structure was a part of the analysis process, since the entire context and meaning of the participants’ words must be taken into consideration. A certain level of interpretation was also necessary in this phase. After this back-and-forth movement, adjustments in the coding and category structure were made when needed. Since the aim was to describe the manifest experiences of the participants, the analysis was terminated when reaching to main categories at a descriptive level with a low degree of interpretation instead of themes that has a higher degree of interpretation on a more abstract level [39].

**Table 2:** Example from the qualitative content analysis process

Meaning unit	Condensed meaning unit	Code	Subcategory	Category	Main Category
I felt completely safe, and it was very important that they asked how I felt, and what I felt was wrong and the problem, that I could explain when I started to feel worse, and why	I felt safe because they asked me how I was doing and I could explain	Safe that they asked, and I could explain	I felt seen, listened to and taken seriously	I was in focus and a contributing part	We were a team of three
No, I … you've never experienced it (videoconsultation), not like that in a hospital, but she was focused on me, and it was calm and quiet in the room and everything. No, I think it was good. If they had only had eye drops in the community hospital, then I would have been super satisfied. [laughs]	I have never had a video meeting in a hospital, but the physician was focused on me, and the room was calm and quiet. If they only have had the eye drops here in the community hospital, I would have been super happy	Dr. was focused on me, calm and quiet in the room. Good! If they had the medicine locally, I would have been very happy	The GP being on the screen worked just as well Long wait and more steps in the care chain	The GPs attendance was important VC could not meet my needs	We were a team of three VC was a two-sided coin

Two interviews were coded and categorised independently by two researchers (LÄ, AEL) and discussed several times to validate the analysis [40]. The remaining 10 transcripts were analysed by the first author and subject to further discussions with another researcher (AEL). To increase credibility, all categories were then discussed with the research team (LÄ, AEL, HD, JC). Two of the authors (HD, JC) do not live in a rural context but have experience conducting research in rural areas and read the complete transcripts several times. This step was taken to address dependability, as the researchers’ pre-understandings and interpretation affect the study results [41]. Disagreements were solved in the research team, and agreement was reached on the final results. To further confirm the study’s reliability, a selection of quotes was used in the results. The quotes have been translated by LÄ from Swedish and reviewed by another researcher (HD). During the analysis the different theoretical lenses in the research team was reflected upon and the researchers different living and working conditions in urban Stockholm, and rural Storuman was especially noticed and found to widen the perspectives.

## Results

Participant characteristics are presented in Table 1. The analysis resulted in two main categories, five categories and 20 subcategories (Table 3). The quotes for the subcategories can be seen in Table 4. The first main category: *We were a team of three* includes the categories: *The RN was the hub*, *The GP’s attendance was important,* and *I was in focus and a contributing part*. The second main category, *VC was a two-sided coin* includes the categories *VC was surprisingly well-functioning and smooth,* and *VC could not meet my needs*.

**Table 3:** Overview of the study categories

	**Main Category 1:** We were a team of three			**Main Category 2:** VC was a two-sided coin	
**Category**	The RN was the hub	The GPs attendance was important	I was in focus and a contributing part	VC was surprisingly well-functioning and smooth	VC could not meet my needs
**Subcategory**	The RN was central to communication The interaction between RN and GP worked well The RN's competence was sufficient	The GP being on the screen worked just as well The GP’s expertise was a must	I felt seen, listen to, and taken seriously Not being alone and, getting the right help feels safe I was involved in the planning and got to hear everything I can tell you what happened and how I feel I did what I could	An effective care chain The visit wasn’t stressful, and I was not a burden Healthcare was accessible and travelling was reduced Sound and image were good enough for the consultation Both good and bad to know each other in emergency visits	Long wait and more steps in the care chain Difficult to see, action, and assess with the GP on video Prefer to meet face-to-face and make better eye contact Staff couldn't handle the technology and it was failing Lack of information and accessibility

**Table 4:** Representative quotations to subcategories

Main category	Category	Subcategory	Quotation
We were a team of three	The RN was the hub	The RN was central to communication	**Q.1** “*After I had talked to the doctor, the RNs talked to me and explained it to me more thoroughly” (P15)*
		The interaction between RN and GP worked well	**Q.2***“(the GP) was good at explaining, and if (the RN) didn't understand … well, then (the GP) explained how it would be, or what it should look like, so I thought it went well” (P14)*
		The RN's competence was sufficient	**Q.3** “*(the RN) was so thorough. (The RN) had to measure the pressure and was very good at it, and (the RN) said: "I'm not experienced with this, but I've studied and watched and so on", but (the RN) knew it.” (P13)*
	The GPs attendance was important	The GP being on the screen worked just as well	**Q.4***“it felt good to having talked to a GP as well, even if it was on the TV screen. You don't have to be physically in the room …”* (P12)
		The GP’s expertise was a must	**Q.5***”but then when (the GP) had seen the wound and saw that it was very deep, (the GP) realised that it is possible … you have to do it in a different way” (P2)*
	I was in focus and a contributing part	I felt seen, listen to, and taken seriously	**Q.6***“… and I got to explain that I had a general feeling of illness in my body, and they took it seriously,… it was me, the RN and the GP, and they both listened”* (P1)
		Not being alone and, getting the right help feels safe	**Q.7** “No, but I thought (the GP) … (the GP) was so secure and so calm here in X, and this RN too. I felt safe all the time. I felt that they were doing the right thing (P13)
		I was involved in the planning and got to hear everything	**Q.8** “Well, I think I was part of the planning, because we planned to wait until the next day, because I was given intravenous medicine, and to go home and see how it developed during the night. So yes, I was definitely part of the planning, because if I had chosen to go to the hospital, I would have been allowed to do so”* (P12)*
		I can tell you what happened and how I feel	**Q.9***“Well, I have to be honest. I have to say how it is, how it feels, what I think, how I experience it. No, they can't read minds.”* (P4)
		I did what I could	**Q.10***“I understood how to make (the GP) see the wound on the screen, so (the RN) never told me how to do it, but I just saw it on the screen”* (P2)
VC was a two-sided coin	VC was surprisingly well-functioning and smooth	An effective care chain	**Q.11***“…and above all I got help quickly… (the RN) took blood samples and such things beforehand…, checked my temerature and made a phone call, and then it was as good as done. So I thought it was great”* (P1)
		The visit wasn’t stressful, and I was not a burden	**Q.12***“Yes, I thought that now she had the time for me, and I didn't feel any pressure to: "Hurry up and say what you have to say". It felt like this was my moment on earth”* (P12)
		Healthcare was accessible and travelling was reduced	**Q.13***“The alternative was to drive somewhere indefinitely. In the dark. So if you compare it to the alternative, I think this is much better”* (P4)
		Sound and image were good enough for the consultation	**Q.14***“No, it worked quite well, you could hear them clearly, the picture screen was good,… the connection was good and there were no bigger problem to hear what he said”* (P7)
		Both good and bad to know each other in emergency visits	**Q.15***“Maybe sometimes, but it depends more on what symptoms you have…but at the same time in this emergency visit I don't know if it would not matter much”* (P14)
	VC could not meet my needs	Long wait and more steps in the care chain	**Q.16***” if you had been able to meet (the GP) at the community hospital, perhaps this referral to (the hospital) would have been a little quicker, so that I might have been able to go sooner. I had to wait (several days) before I came to (the hospital)” (P7)*
		Difficult to see, action, and assess with the GP on video	**Q.17″***The thing that perhaps made it difficult for the GP in a way is that … yes, but when it's the throat, (the GP) can't see…”* (P14)
		Prefer to meet face-to-face and make better eye contact	**Q.18***“…you know when you meet each other in person, then … you keep eye contact, and you see if the other person is looking at something else. I couldn't see that in her eyes, if she was looking at something else.”* (P 4)
		Staff couldn't handle the technology and it was failing	**Q.19***“ it was like a close up of my face so when we tried to talk to the doctor on the screen, it was just like … but I think it was more that (the RN) didn't know how to kind of change the camera.”* (Patient 8)
		Lack of information and accessibility	**Q.20***“And they are not able to help me because there was no one … there was a GP on call in (the community hospital A) and it was like either I have to drive to (the hospital) and get an X-ray there, or … there was no one in (community hospital B) who could X-ray me, there was no GP there. The RN said: "It's not worth it for you to come here, because it's not worthwhile for me to look at it and send you off. There's no point, it's just troublesome for everyone." (P4)*

### Main category 1: We were a team of three

#### The RN was the hub

Throughout the emergency visits at the community hospitals, the RNs were at the centre of communication with the patients (Table 4; Q1). Before connecting to the GP, the RN sometimes spoke to the GP over the phone, and they decided whether to conduct a VC. When the GP had disconnected from the VC, the patients were aware that the RNs could explain further in detail what was said in the VC and the plan for their care in more detail. Patients experienced that in cases where the GP was stressed, the RNs were able to step in and communicate important information. In some cases, patients perceived that the information was given to them through the RN.

When the GP was present on the video screen, there was an interaction between the RN and the GP that patients believed worked well (Table 4; Q2). The GP was able to offer explanations and give instructions, both regarding the use of equipment and examination of the patient.

Patients experienced that the RNs working in the emergency room at the community hospitals had the competence needed to perform the actions the GP would have performed if he/she would have been present in the room (Table 4; Q3). Patients described the RNs as skilful, trustworthy and thorough when performing the examinations. Patients felt that they were treated well and often felt that the RNs were as competent as the GPs. To be the hub of the visit, RNs need to be able to perform basic care procedures, such as collecting samples and dressing wounds. But in this study, RNs also needed to have more advanced knowledge to perform assessments of the heart and lungs with a stethoscope and to suture minor wounds.

* “…and then the GP got to look at my leg, and … well, got to see when (the RN) palpated it, …ordered to X-ray it, and then (the GP) would look at the X-ray image and we would get in touch later, and then they X-rayed it on the spot, and (the GP) looked at the image.”* (Patient 4).

#### The GPs attendance was important

Meeting with the GP on the screen was a positive experience for the patients. They expressed that it did not matter that the GP worked remotely and that it was as good as a face-to-face consultation (Table 4; Q4). Some patients even expressed that it felt as though the GP was in the room. The patients experienced that the GP was easy to open up to when they connected over VC. They could communicate well and they felt that the VC was personal even if they had never met the GP before. The GP´s behaviour played a major role in the experience of care, and the patients expressed feeling calm, that they were taken seriously and were included in the care visit.

Although the RNs played a major role in the emergency room, patients expected to meet a GP when seeking emergency care. Patients felt that a GP´s competence was essential, particularly when the RN was uncertain and in need of instruction and encouragement (Table 4; Q5). Patients described the GP´s presence as important when more assessment was needed and when the GP needed to consult further with a specialist care unit. Patients had the impression that the GP was responsible for making decisions on further treatment or testing. The patients experienced a sense of safety and happiness when they were able to see and speak to the GP themselves instead of communicating exclusively through the RN over the phone. Patients stated that they trusted the GPs to know what to do and how to help them.

*“It felt like there was a GP making a decision, seeing how I was feeling before they said: "I would go home until tomorrow morning." And it felt safer”* (Patient 12).

#### I was in focus and a contributing part

The patients experienced that the VC led to feelings of inclusion. They felt that the focus was on their needs in the emergency room and that they were taken seriously and acknowledged, which was important for a sense of safety in an uncertain situation (Table 4; Q6). Some patients felt that this sense of acknowledgement was even more important in a VC. Patients felt safe when they were accompanied by someone and believed the healthcare professionals offered them the right help (Table 4; Q7).

The continuous presence of the RN and the GP on the screen was seen as a positive aspect of the VC, as the patient got to hear everything, felt involved and received the same information as the care providers (Table 4; Q8).

*“It never happened that they left the room, do you understand what I mean? Sometimes … if you seek emergency care somewhere, and you're sitting in a room and the GP comes in and the GP leaves the room, and then they talk outside the door with a RN or with lab staff … "Can you do this or that? Should you do this or that? Or what do you think?", and so on. But I was there all the time. They had no chance to do anything like that.”* (Patient 4).

In the interaction with the RN and GP, patients felt that it was important that they were honest and provided information about their medical history and the event, as well as to share their thoughts and feelings during the ongoing emergency situation. The use of VC sometimes meant that they needed to tell their story twice, first to the RN and then to the GP, but this was not seen as a problem. On the contrary, the patients wanted to tell their stories to the GP themselves (Table 4; Q9).

One way for patients to contribute was to independently show the GP the injury with the help of a video that appears on the GP’s screen. In some cases, the patients’ own responsibility for making travel arrangements to, and between care units contributed to feelings of anxiety, both before the VC and after. Especially in cases where a VC alone was not enough, and the GP wanted to meet the patient in person at another community hospital. The patients also tried to do what they could to dress their own wounds, waiting for pain to pass and taking medication before seeking emergency care (Table 4; Q10).

### Main category 2: VC was a two-sided coin

#### VC was surprisingly well-functioning and smooth

The patients described this form of emergency care as more effective than they had expected. The patients simply needed to call to explain their condition and then they could come to the community hospital immediately, which was thought to be within the travel time of 20–60 min (Table 4; Q11). When they arrived at the community hospital, the RN welcomed them at the door and started the assessments, sampling and preparation for the VC with the GP. Many of the patients stated that contacting the healthcare providers was very easy, and they were surprised how quickly they were able to speak with a human, not an answering machine, and receive a consultation with a GP, even after office hours. This was unexpected based on the patients’ previous experiences of emergency care visits. Several patients emphasized the ease of the emergency VC and the fact that the RNs and GPs were focused on them.

*“When you’re in a video call like this, that's what you do. I kind of got the feeling that the focus was on me and my hand, and maybe the fact that he wasn't physically present even was a positive thing, [laughs] because then he had that time slot, and I was the one that mattered. That's how I felt.”* (Patient 1).

They also felt that they were not treated as a burden. Instead, the patients were treated well, even if the staff sometimes needed to work overtime (Table 4; Q12).

In cases where patients stated that the VC worked well, they saw it as a positive that they needed to travel less (Table 4; Q13). In these cases, they also experienced satisfactory picture and sound (Table 4; Q14). The patients were often familiar with the healthcare staff, which was either perceived as a positive or insignificant in the acute situation (Table 4; Q15).

#### VC could not meet my needs

Some of the patients stated that the VC became an unnecessary step in the care chain, especially when the RN did not have the right competence or did not feel confident providing the care that the patient needed. On these occasions, patients had to travel further to see the GP in person, or they were sent directly to the hospital after the VC at the community hospital (Table 4; Q16). Some patients felt that the GP had difficulty performing the necessary assessments over VC and that sometimes, it was not possible at all. Some patients also found that the GP had difficulties recognizing when they were in in pain (Table 4; Q17).

*“Well, if you just compare it with the fact that there was a RN in the room, and (the GP) on the TV, I would say that … the RN who was manipulating the thumb noticed quite quickly that: "It's quite painful for the patient to move his thumb." But the (the GP) on the computer screen is just looking at the thumb.”* (Patient 7).

Some patients expressed that they would have preferred to see the GP face-to-face. Some of the reasons the patients gave were the lack of eye contact with the GP and trouble seeing what the GP was looking at on the screen. This felt more impersonal and decreased the feeling of connection with the GP (Table 4; Q18). Technical problems occasionally occurred during the VC, and the health professionals did not always know how to use the technology properly. This was particularly bothersome for some patients, and the VC became an unpleasant experience (Table 4; Q19). Not knowing where to turn for emergency care and the location of GPs on-call made some patients feel insecure, and they were also concerned about the lack of information about test results (Table 4; Q20).

## Discussion

The aim of this study was to explore patients’ experiences of emergency care via VC in northern rural Sweden. The patients’ feelings of inclusion in a team together with healthcare professionals is one of the main categories, “We were a team of three”. Patients felt that VC functioned well because of the RN’s professionalism and competence to be the hub of the visit. These patients also appreciated the ability to interact with the GP via video. Further, the VC was seen as a two-sided coin (the second main category), working smoothly and efficiently for some patients, but unable to meet the needs of other patients. In cases where emergency VC was seen as insufficient, it was often due to difficulties related to remote assessment by the GP in combination with a lack of competence among RNs. In several cases, this led patients to describe VC as inconvenient and requiring more steps in the care chain, as the patient sometimes needed to travel further to meet the GP face-to-face.

### Competent local RN – a must

Patients expressed that they were treated well and often attributed this to their trust in the RN, who they saw as skilful and competent. This is in line with research that found that the relationship with the local RN is especially important in digital care, because being emotionally close to the RN can give patients feelings of familiarity and presence despite the use of digital care [21]. The central role of the local RN could be magnified as the RN is the only person physically present in the room [42]. Some studies also show that the rural context may have an impact, making the relationship between the patient and healthcare professionals even more important. Patients often describe RNs in rural communities in very familiar terms, almost as friends [43], and the importance of building trust based on relationships and social networks has been highlighted as typical for the rural context [21, 27]. Thus, our findings stating that some patients were concerned about the long distance and need to make travel arrangements by themselves to access emergency care indicate the importance of having healthcare professionals with knowledge about the rural context who can support patients with e.g. practicalities connected with long distances, such as the need to buy food, access to toilette and rest when travelling and waiting can be long. The partnership between patients and practitioners in rural areas can be more challenging, since rural practitioners often have medical training that cannot be applied in the rural context [22]. Although digital technology makes it possible to access medical expertise from other geographical locations [44] to address staffing shortages, there are risks associated with further reliance on VC, as healthcare staff who work and live in urban centres will likely have less knowledge of the rural context. This could affect the quality of healthcare negatively for the people living in these areas, which has already been pointed out [22, 45]. Further research is needed to investigate outcomes when healthcare professionals offer VC to rural populations and how the relationship between healthcare professionals and patients is affected by digital health services.

Some patients expressed that VC failed to meet their needs. On these occasions, it was sometimes due to a lack of competence and experience on the part of the RN. As a result, the patient needed to travel to see the GP face-to-face, which required more time and created more steps in the care chain, while increasing inconvenience, insecurity and effort. This indicates that education specifically designed for these settings, which includes the use of emergency VC in community hospitals, needs to be of high priority. Furthermore, the need for context-specific training has also been recognized in previous research [27, 36, 46–48].

### VC in emergency care needs to be improved

In this study, some patients experienced VC as an additional step in the care chain because the right assessment could not be done remotely by the GP or the RN, even for relatively minor injuries. The result also highlights patient experiencing that the GP could not always recognize how they were feeling from behind the screen and that the lack of eye contact was problematic. Taken as a whole, this made the VC a less positive experience for some patients. Research has shown that telemedicine is an effective way to manage a broad spectrum of acute conditions when used together with portable devices, such as electronic stethoscopes or digital otoscopes [49]. Peripheral units with high-resolution images of eyes, throat or skin, as well as audio from lung sounds, can be used to collect patient vitals and send information to a physician digitally, thereby enabling remote diagnostic decisions [49]. VC in emergency care could increase the capacity to manage patients locally and reduce unnecessary transfers [50]. It has already resulted in better service use patterns, improving diagnosis by bringing expertise into fist-line management [51]. It is likely that in the future, emergency VC can further improve in quality. Investing in better digital equipment in the emergency rooms of rural community hospitals could increase the ability of the GP to perform remote assessments. This would not only improve the patient experience, it would also assist the RN and GP maintaining their roles in nursing and medicine when the GP can examine the patient more independently.

In this study, patients are experiencing emergency VC to have both positive and negative effects, which shows a broad and individual variation of experiences in our results. A systematic review exploring patients’ and caregivers’ satisfaction with telehealth, particularly through VC, found consistent evidence that telehealth has an overall positive impact on satisfaction [10]. Interestingly, a systematic review on patient satisfaction with telemedicine in rural and urban acute care settings showed that satisfaction is a complex mix of expectations and experiences, but is nevertheless an important indicator of the quality of the service [49]. Convenience is a major determinant of quality for the patient and a strong predictor of satisfaction with telemedicine. This is in line with our interpretation of the source of the contradiction in our results. Some patients in our study were surprised by how effective VC care was and the ability to have contact with a GP after hours. They were also very satisfied with the convenience of VC, as they did not need to travel long distances. Others expected to meet a GP face-to-face and were generally disappointed with the VC. Different expectations may explain the contradictory experiences in the results.

### Seeing the GP is important

The findings from our study show that the patients experience the GP to have an important role in their emergency care as they had the expertise and made the decisions. The difference between attending on a screen or face-to-face did not seem to be of importance in most cases, but the ability to have direct contact made them feel more secure and even happy. A parallel can be drawn to research studying patients’ experiences of using VC for psychiatric emergencies, where direct contact with the psychiatrist made the patients feel calmer and more certain that the right assessments and decisions were made [24]. Many patients in our study stated that VC was effective and not stressful. Some stated that the RN and the GP were very focused on them. One patient felt that the VC increased the focus of the GP, as there were less things requiring the GP’s attention. These findings are valuable, since the study provides insight into positive ways to further develop and improve emergency VC. Research studying how patients participate in interactions with their GP during VC in other settings has found that patients show sustained engagement in the conversation, which indicates that VC provides a communicative context that allows patients to participate and engage in new ways [52]. In these studies, patients felt that undivided attention from clinicians made it possible to build relationships, however, clinicians found it challenging to maintain high-quality relationships without face-to-face consultations [53]. This is in line with research investigating simulated emergency care via VC with a GP in northern rural Sweden, where GPs rated shared decision-making lower than RNs which may imply that the GP-patient relationship is especially vulnerable with the GP via VC, something that is worrying since shared power already is low in general emergency care settings. This study also showed that health care professionals rated patient’s opportunities to ask questions lower during VC [54]. Participation and relationships have been shown to be important for good quality care via VC, but the communication component needs to be given special attention because of the way VC differs from a face-to-face consultation [55]. In contrast, research that compared the content and quality of VC and face-to-face consultations found that clinicians were more engaged during face-to-face consultations and that this format was a more effective way to build a partnership [14]. Patients in previous research have also stated that they prefer to meet face-to-face if they have a choice [12, 13, 56–58], and this was also expressed by some patients in our study. However, well educated, young adults believe that VC is as good as a face-to-face consultation. Still, research is needed to better understand the perspectives of other groups [9].

In the present study, patients stated that it was important for them to see and speak to the GP themselves instead of only through the RN over the phone. This is an important result, as it raises the question of whether VC is as good, or better, than a traditional telephone call, since this could be another way to remotely consult the GP from the emergency room. Further, research comparing telephone consultation and VC has been done to investigate this very question [14, 58–62]. One RCT showed that VC in prehospital emergency care did not increase the proportion of patients treated and remaining in their home community compared to a phone call, nor did it increase patient satisfaction [62]. In contrast, a systematic review comparing VC and telephone consultation in healthcare delivery found that in some cases, VC was superior to a phone call [61]. This shows that VC, as a two-sided coin, still needs to be explored further, but there are clearly benefits to be realized.

### Strengths and limitations

This study is one of the first studies done in this area. The included patients age ranged from 18–89 years and had a good gender balance, which contributes to the credibility of the study, as it adds a richer variation of experiences [38]. The patients were recruited by the RNs in the emergency rooms, which could be seen as a risk of bias if dissatisfied patients were not invited to participate. However, the results clearly show a variation of both positive and negative experiences. To investigate decline to participate, efforts were made to gather data on the total number of VC emergency visits during this time period, but this data is not logged in the system or manually in any reliable way. During recruitment, it was not noted if any patients declined participation, which is a limitation of the study.

Seeking agreement among the authors through discussions of the result and analysis process, as well as quotes in the reporting of the study, is ways to strengthens the study’s credibility that has been applied by the authors [38]. The data collection period was long due to the limited size of the patient population, the occasional use of telephone between RNs an GPs instead of VC, and due to the high workload for the RNs in the community hospitals. But we have no reason to believe that the dependability of the research was affected by this. Rigorous descriptions of context, setting, participant characteristics, analysis and results have been made to facilitate the interpretation and the transferability of the results. Experiences of emergency care with VC are expected to be transferable to other groups, to some extent, even if such interpretations should be done with caution. To further strengthen the trustworthiness of the study, the manifest content has been sought with a low degree of interpretation, resulting in categories with quotations. Information is presented on the authors and their different backgrounds in order to ensure transparency about personal history, which inevitably influences interpretations.

## Conclusion

Patients in rural areas experience emergency care via VC as a visit where they were in the center of attention and as included in the team. But VC also has disadvantages e.g. not allowing physical examinations by the GPs and decreased feelings of personal connection. The experience seems to be dependent on the individuals’ expectations and circumstances. Appropriate use of VC should always be determined depending on the unique needs of the patient. The key role of RNs during the emergency care via VC was clear and proper training in emergency care and rural context would further strengthen the nursing profession. Technical devices are needed to support GPs to perform assessments and reach an appropriate decisions, thus fulfilling their role as responsible medical professionals independently and remotely. Patients expected the GP to contribute with their competence and communicate directly to the patient. If patients can choose, they prefer to meet face-to-face instead of in VC. This result highlights the benefit of VC when it is not possible for the GP to be on site, since the patient experiences of seeing and talking to the GP themselves is truly valuable. It is also a reminder that the possibility to choose should be pursued. Emergency care via VC in rural areas can likely be more effective and increase patient satisfaction if these requirements are met. Future research is therefore crucial to explore how healthcare should be organised to have the flexibility for individual needs of patients and how different roles in emergency care via VC can be supported to ensure the best possible patient experience in rural areas. A possible implication of this study is to develop education in emergency care via VC and in the technology available that could increase the quality of emergency care via video.

### Supplementary Information

Supplementary Material 1.

## Data Availability

Data can be found in Tables. The data generated and analyzed during this study are interviews related to people’s health and therefore personal data. The setting of data collection is sparsely populated areas which makes data extra privacy sensitive with a risk of identifiable information. Due to this, data from this study is not publicly available.

## Data availability

No datasets were generated or analysed during the current study.
