# Supplementary material for: Emergency care via video consultation: interviews on patient experiences from rural community hospitals in northern Sweden
Source: Int J Emerg Med. 2024 Sep 3;17:109. doi: 10.1186/s12245-024-00703-4 (PMC11370045; doi:10.1186/s12245-024-00703-4)
Supplement: Supplementary file 1 — Supplementary Material 1. [file 12245_2024_703_MOESM1_ESM.docx]

**Appendix 1.** Interview guide

| Interview guide to patients in acute care VC visits |
| --- |
| **Background questions:** Age, Gender, cause of care contact, Born and raised in, Familiarity with digital solutions (such as social platforms, Skype, Bank ID).   1. **How do you think health care works for you in general today?**  - What are the needs? Are they met?  1. **Tell us about your visit to the emergency room.**  - What it is like, not to be in the same room as all the healthcare professionals, but with GP on video?  1. **How did you feel about the information you received from the staff?**  - Was it sufficient? - What felt important to be informed about/not be informed about?  1. **Describe the treatment you received from the healthcare team**.  - How did you communicate with each other? - Were you involved in the conversation? - Which person(s) did you get in touch with, in what way?  1. **In what way did the healthcare staff listen to you and what impact did this have?**  - How did it feel? Why do you think it felt like that? - Did you wish that it had gone differently? How?  1. **How would you describe that you were part of what concerned your care during the visit?**  - How would you like to be involved in the planning of your care?  1. **Can you tell us if anything during the visit made you feel safe, or not safe?**  - Why do you think this was the case?  1. **What do you think you can contribute with in the care visit?**  - What is important for you to contribute with?  1. **How did you come in contact with healthcare when you became acutely ill?** 2. **What is important to you when you have contact with health care?**  - What is good quality care for you? - Does it apply to both emergency, and planned visits? Why?   **Summarize the questions. Is there anything else you would like to add? Thank you very much!** |
